# Identification of ATP binding residues of a protein from its primary sequence

**DOI:** 10.1186/1471-2105-10-434

**Published:** 2009-12-19

**Authors:** Jagat S Chauhan, Nitish K Mishra, Gajendra PS Raghava

**Affiliations:** 1 Institute of Microbial Technology, Chandigarh, India

## Abstract

**Background:**

One of the major challenges in post-genomic era is to provide functional annotations for large number of proteins arising from genome sequencing projects. The function of many proteins depends on their interaction with small molecules or ligands. ATP is one such important ligand that plays critical role as a coenzyme in the functionality of many proteins. There is a need to develop method for identifying ATP interacting residues in a ATP binding proteins (ABPs), in order to understand mechanism of protein-ligands interaction.

**Results:**

We have compared the amino acid composition of ATP interacting and non-interacting regions of proteins and observed that certain residues are preferred for interaction with ATP. This study describes few models that have been developed for identifying ATP interacting residues in a protein. All these models were trained and tested on 168 non-redundant ABPs chains. First we have developed a Support Vector Machine (SVM) based model using primary sequence of proteins and obtained maximum MCC 0.33 with accuracy of 66.25%. Secondly, another SVM based model was developed using position specific scoring matrix (PSSM) generated by PSI-BLAST. The performance of this model was improved significantly (MCC 0.5) from the previous one, where only the primary sequence of the proteins were used.

**Conclusion:**

This study demonstrates that it is possible to predict 'ATP interacting residues' in a protein with moderate accuracy using its sequence. The evolutionary information is important for the identification of 'ATP interacting residues', as it provides more information compared to the primary sequence. This method will be useful for researchers studying ATP-binding proteins. Based on this study, a web server has been developed for predicting 'ATP interacting residues' in a protein http://www.imtech.res.in/raghava/atpint/.

## Background

Adenosine-5'-triphosphate (ATP) is an important molecule in cell biology as an energy molecule and coenzyme. This molecule interacts with large number of proteins during cellular activities and plays a crucial role in various biological reactions. ATP binding proteins (ABPs) have a binding site that allows ATP molecule to interact. This binding sites is a micro-environment where ATP is captured and hydrolyzed to ADP, releasing energy which is utilized by the protein to "do work" by changing the shape of the protein and/or making the enzyme catalytically active. These proteins are powered by the hydrolysis of ATP and convert this chemical energy for mechanical work [1]. Many ATP Binding proteins are transmembrane proteins and responsible for transport of a wide variety of substrates (e.g. lipids, sterols) across extra and intracellular membranes [2]. In summary, ATP binding proteins have important roles in membrane transport, muscle contraction, cellular motility and regulation of various metabolic processes.

Thus it is important to identify ATP binding proteins and 'ATP interacting residues' in these proteins. The experimental identification of residues that interacts with ATP in a protein is costly and time consuming. Thus there is need to use alternate techniques such as computational technique, which have been used successfully for predicting function of proteins [3-12]. In past, methods have been developed for the prediction of polynucleotide (DNA/RNA) interacting residues [8,13,14]. Saito et al [15] developed a general method for predicting nucleotide-binding sites in a protein, which successfully predicts 31% ATP binding sites (not ATP interacting residues). To the best of our knowledge, no prediction method has been developed for detecting specifically the residues interacting with ATP from a protein sequence. Thus, there is a need to develop method for predicting 'ATP interacting residues' in a protein in order to understand protein-ATP interaction.

In this study, a systematic attempt has been made to develop a highly accurate and reliable method for predicting 'ATP interacting residues' in a protein. Initially, Support Vector Machine (SVM) based models have been developed using proteins sequence. In the past, it has been shown that the evolutionary information provided more information [16,17] than protein sequence, thus we have also used evolutionary information in the form of PSSM profile for developing a prediction method. All the models developed in this study were evaluated using five-fold cross validation technique.

## Methods

### Datasets

We extract 360 ATP binding protein chains from SuperSite encyclopedia [18]. After removing the redundant sequences using the program CD-HIT, a total of 267 non-redundant PDB chains were obtained where no two sequences have more than 40% identity. In the next step, we examined these proteins using software Ligand Protein Contact (LPC) [19] and remove those proteins, which are not ATP binding proteins according to LPC. Our final dataset have 168 non-redundant ATP binding protein chains, available at http://www.imtech.res.in/raghava/atpint/atpdataset

### Five-fold cross-validation

Evaluation of a newly developed method is a major challenge for researchers. One of the commonly used techniques for evaluating a model is jack-knife or leave-one-out cross-validation (LOOCV) [4,20,21]. In this technique one sequence is used for testing and remaining sequences for training, this process is repeated in such a way that each sequence is used once for testing. Though this is the best technique for evaluation, it is time consuming and computer intensive. Thus, we have used 5-fold cross-validation in this study where sequences were randomly divided into five sets. One set was used for testing and the remaining four sets were used for training. This process was repeated five times in such a way that each set was used once for testing [9,22]. The final performance was obtained by averaging the performance of all five sets.

### Pattern or window size

We have generated overlapping patterns (segments) of different window sizes from 7 to 25 for every ATP binding protein sequences. If the central residue of the pattern was a 'ATP interacting residue', then we assigned the pattern as positive pattern (ATP interacting) otherwise it was assigned as negative pattern (non-ATP interacting). To generate the pattern corresponding to the terminal residues in a protein sequence, we have added (L-1)/2 dummy residue "X" at both terminals of protein (where L is the length of pattern) [9]. As an example, for window size 17, we have added 8 "X" before N-terminal and 8 "X" after C-terminal, in order to create M patterns from sequence of length M [16,17]. Finally we have obtained a total of 3056 unique windows/patterns of length 17 out of 3082 ATP interacting residues.

### Support Vector Machine (SVM)

In most of our studies including this one, we have implemented SVM using SVM light [23], which is freely downloadable package from http://svmlight.joachims.org/. SVM is a machine learning approach based on structural risk minimization principle of statistics learning theory [24]. The main reason of using this package frequently by us is that it allows implementing various kernels and parameters.

### Position Specific Scoring Matrix (PSSM)

In this work, PSSM profiles were generated using PSI-BLAST [25] where a protein sequence was searched against SWISS-PROT dataset using E-value cut-off of 0.001. This profile contains the probability of occurrence of each type of amino acid at each position along with insertion/deletion. Hence, PSSM is considered as a measure of residue conservation in a given location. This means that evolutionary information for each amino acid is encapsulated in a vector of 21 dimensions where the size of PSSM matrix of a protein with *M *residues is 21 × M, where M is the length of the target sequence, and each element represents the frequency of occurrence of each of the 20 amino acids and one dummy amino acid "X" at one position in the alignment [16].

### Structural feature

In this study we have used following seven important structural feature as SVM input feature -

#### Hydrophobicity

The hydrophobicity effect is often a major contributor of binding affinity between a protein and its ligand. All Hydrophobicity calculations were obtained from Fauchère and Pliska scale [26].

#### Beta-Sheet

Many nucleotide-binding proteins having a P-loop or phosphate-binding loop, is an ATP binding site motif. It is a glycine-rich loop preceded by a beta sheet. Thus the Beta-Sheet may be important feature in the ATP binding protein. It is obtained from Chou and Fasman scale [27].

#### Polarity

Polarity is a separation of electric charge leading to a molecule having an electric dipole. It results from the uneven partial charge distribution between various amino acids in a protein. We have used Grantham R polarity scale values [28].

#### Solvation potential

The solvation potential is an important parameter of proteins that gives an idea about the preference of amino acid residues to be exposed to solvent or buried in the interface. For calculation of solvent potential for each amino acid, we have used *Jones et al *scale [29].

#### Residue interface propensities

The residue interface propensity is an important feature of protein binding sites that shows the propensity of each amino acid residues in the interface area. Residue interface propensities for each of the 20 amino acids were computed from Jones and Thornton [30].

#### Net charge

The surface of a protein has a net charge that depends on the number and identities of the charged amino acids, and on pH. At a specific pH the positive and negative charges will balance and the net charge will be zero. Net charge of amino acid obtained from Klein et al [31].

#### Average accessible surface area

The accessible surface area is the surface area of a protein that is accessible to another protein or ligand. The average accessible surface area scale values of each amino acid were obtained from Janin et al [32].

All the above features, parameter, scale values were taken from http://www.genome.jp/aaindex[33] and used as input features in SVM.

### Evaluation Parameter

For evaluation of the performance of methods, we have used standard parameter that is routinely used in this type field. Following is a brief description of the threshold dependent parameters which was used for evaluation.

#### Sensitivity

This parameter allows computation of the percentage of correctly predicted ATP interacting residues.

#### Specificity

This parameter allows computation of the percentage of correctly predicted non-ATP interacting residues.

#### Accuracy

Percentage of correctly predicted ATP interacting and non-interacting residues

#### Matthews's Correlation Coefficient (MCC)

It is the statistical parameter to assess the quality of prediction and to take care of the unbalancing in data. An MCC equal to 1 is regarded as a perfect prediction, whereas for a completely random prediction this value becomes 0.

Where TP is the number of correctly predicted ATP interacting residues, TN is the number of correctly predicted non-interacting residues, FP is the number of non-interacting residues predicted as interacting residues and FN is the number of interacting residues wrongly predicted as non-interacting.

All the parameters described above are threshold dependent parameters, thus performance of a model depend on threshold. In order to provide the comprehensive view of performance of a model, we have calculated these parameters on different threshold (range from +1 to -1).

#### Area under the ROC Curve (AUC)

All the measures described above have a common drawback that their performance depends on threshold selected. A known threshold independent parameter is Receiver Operating Curve (ROC). It is a plot between true positive proportion (TP/TP+FN) and false positive proportion (FP/FP+TN). We have used SPSS package to plot ROC and calculate AUC.

## Results

### Composition analysis

**Figure 1:**
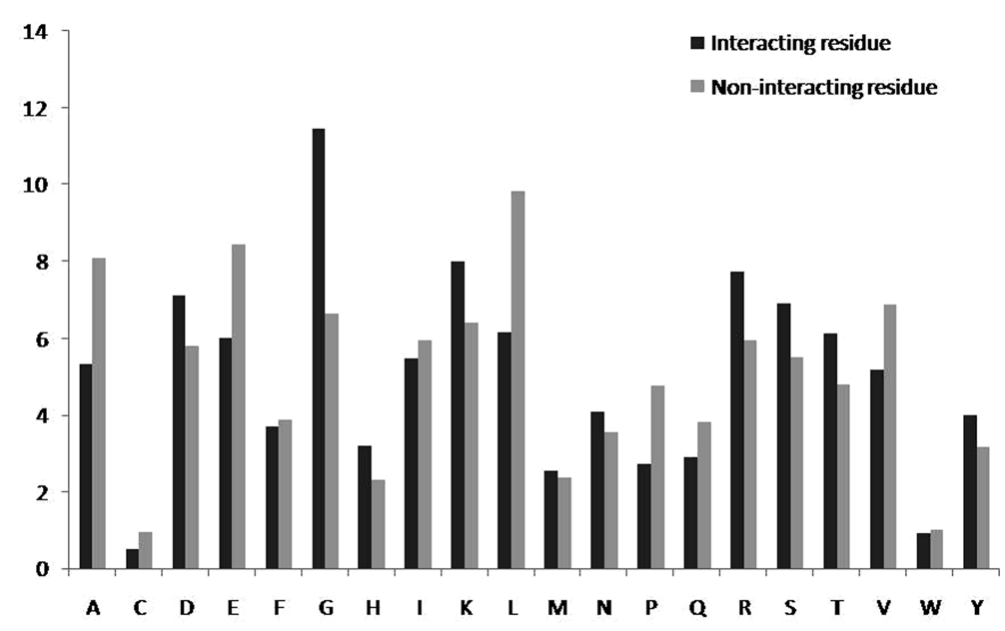
**Percentage composition of ATP interacting and non-interacting residues**.

We have analyzed the composition of interacting and non-interacting residues by computing the amino acid composition of 17 amino acid pattern where the central residue was interacting or non-interacting and observed that the occurrence of Gly and positively charged amino acids Arg, Lys and His were significantly different in ATP interacting residue and non-interacting residues (Figure 1) with respective p-value 0.00186, 0.00862, 0.00254 and 0.07941. It can be inferred that the Gly and positively charged amino acids are important for the interaction with ATP. Beside above residues, significant compositional difference was observed for following residues Leu, Pro, Ala and Val with p-value 0.00511, 0.00011, 0.00049 and 0.02253 respectively (Table S1; see in Additional file 1). It shows that non-polar and hydrophobic amino acid residues such as Val, Leu are important in protein-ATP binding. We have used two-tailed unpaired T-test to check the significant in difference of amino acid compositions in binding and non-binding residues.

### Prediction using BLAST

One of the methods which is routinely used for predicting function of a new protein sequence is BLAST. It is a similarity based method and identifies segments/regions in the query sequences which are similar to the target sequence. This method can be used to predict ATP interacting residues in a protein by searching a query protein against database of ATP-binding proteins. In order to evaluate the performance of BLAST on dataset used in this study, we have searched each ATP binding protein chain against remaining ATP binding protein chains. It was observed that only 71 ATP interacting protein chains showed similarity (BLAST hit) with other ATP binding protein chains. Thus, BLAST cannot be used to predict any ATP interacting residues in 97 ATP binding protein chains out of 168 chains in our dataset. In order to evaluate performance of BLAST on those protein chains which, showed similarity, we randomly picked 10 proteins, which have similarity with other ATP-binding protein chains. Even on these proteins, the performance of BLAST was very poor, where the sensitivity was 44% and the probability of correct prediction was 43.37%. This result suggests that BLAST is not suitable for predicting ATP interacting residues in a protein.

### SVM Modules using single sequence

**Figure 2:**
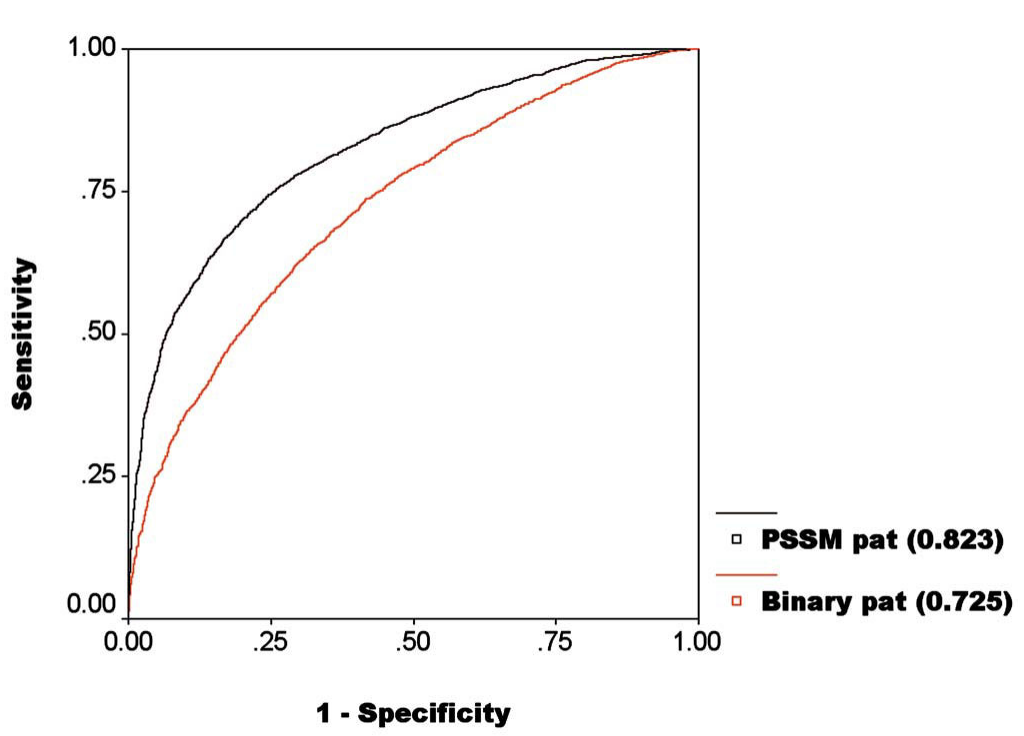
**ROC plot shows performance of SVM modules developed using amino acid sequence and PSSM profile**.

It has been shown in previous studies on nucleotide interacting proteins that they perform best for 17-window size (pattern length) [16,9]. Thus we have used pattern length 17 for developing our prediction model. All possible overlapping peptides of 17 amino acids were generated from ATP binding proteins/chains, a peptide/pattern is assigned ATP interacting or positive if the residue at its center is ATP interacting otherwise it was assigned as negative. After classifying them as positive and negative patterns, they were converted into binary patterns. The peptide of length N was represented by a vector of dimensions N × 21, where each residue is represented by a vector of dimension 21 (e.g. Ala by 1,0,0,0,0,0,0,0,0,0,0,0,0,0,0,0,0,0,0,0,0; Cys by 0,1,0,0,0,0,0,0,0,0,0,0,0,0,0,0,0,0,0,0,0); contains 20 amino acids and one dummy amino acid "X". Our SVM module predict a score for each residue in protein (in range of -1.0 to 1.0), we define a threshold to discriminate ATP interacting and non-interacting residues. The performance of SVM module developed using a single sequence for window size 17 is shown in Table 1. We have also tried various window sizes from 7 to 25 residues and observed that 17 window size patterns gave better performance (Table 2). We have achieved 66.25% accuracy with minimum difference between sensitivity and specificity and MCC 0.33 by 17 window patterns (Table 1) at threshold 0.0. Normally we select a threshold where sensitivity and specificity are nearly equal, in order to make the balance between sensitivity and specificity. The performance of SVM model for window size 17 using single sequence is shown in Figure 2. We have achieved AUC 0.725 which was significantly better than random (AUC 0.5).

**Table 1:** The performance of SVM model (learning parameter: g: 0.1 c: 2 j: 3) using amino acid sequence (The SVM parameter g (in RBF kernel), c: parameter for trade-off between training error & margin, j: cost-factor)

Thres	Sen	Spec	Accuracy	MCC
-1	100	1.73	50.87	0.09

-0.9	99.87	2.88	51.37	0.11

-0.8	99.67	4.39	52.03	0.13

-0.7	99.25	6.51	52.88	0.15

-0.6	98.36	10.31	54.34	0.18

-0.5	96.89	15.78	56.33	0.22

-0.4	93.75	23.54	58.64	0.24

-0.3	88.94	32.9	60.92	0.26

-0.2	83.4	43.31	63.36	0.29

**0**	**65.53**	**66.97**	**66.25**	**0.33**

0.1	54.6	76.99	65.79	0.32

0.2	43.11	84.98	64.04	0.31

0.3	33.94	91.16	62.55	0.31

0.4	25.7	94.7	60.2	0.28

0.5	18.07	97.15	57.61	0.25

0.6	12.64	98.53	55.58	0.22

0.7	8.71	99.18	53.94	0.19

0.8	6.22	99.54	52.88	0.16

0.9	3.93	99.77	51.85	0.13

1	1.87	99.84	50.85	0.08

(Bold values indicate the point where sensitivity and specificity is roughly equal with maximum MCC.)

**Table 2:** The performance of SVM model using binary pattern of different window size patterns.

Window size	Threshold	Sensitivity	Specificity	Accuracy	MCC	parameters
7	0	60.99	64.47	62.73	0.25	g:0.1 c:1 j:1

9	0	61.25	67.41	64.33	0.29	g:0.1 c:1 j:1

11	0	63.2	64.01	63.61	0.27	g:0.1 c:3 j:3

13	0	63.4	64.81	64.11	0.28	g:0.1 c:2 j:1

15	0	61.62	67.11	64.37	0.29	g:0.1 c:1 j:1

**17**	**0**	**65.53**	**66.97**	**66.25**	**0.33**	**g:0.1 c:2 j:3**

19	0	61.98	69.44	65.71	0.32	g:0.1 c:1 j:1

21	0	60.86	69.96	65.41	0.31	g:0.1 c:1 j:1

23	0	63.65	68.52	66.08	0.32	g:0.1 c:2 j:2

25	0	63.32	70.26	66.79	0.34	g:0.1 c:2 j:1

(Bold values indicate the values where accuracy highest and sensitivity and specificity are roughly equal)

### SVM Modules using evolutionary information

In the past, it has been shown in several studies that evolutionary information gives more information about a protein than single sequence [34,35]. In this study the evolutionary information in the form of PSSM profile has been used for predicting ATP interacting protein residues. The PSSM profile for each sequence was generated using PSI-BLAST where sequence was search against of SWISS-PROT. Each element of PSSM matrix was normalized before using it as an input feature of SVM module. The performance of SVM module that was developed using PSSM, at different threshold, is shown in Table 3. We have achieved maximum MCC 0.51, with accuracy 75.25% at threshold -0.1. These results indicated that the evolutionary information was very important for predicting ATP interacting residues as performance increase significantly from MCC 0.33 to 0.51. The performance of SVM model based on evolutionary information is shown by ROC plot in Figure 2 which indicates an improvement from AUC 0.725 to 0.823 and thereby clearly suggesting that SVM model based on PSSM profile perform better than module based on single sequence.

**Table 3:** The Performance of SVM model (Learning Parameter: g: 0.01 c: 4 j: 1) Using PSI-BLAST Profile

Threshold	Sensitivity	Specificity	Accuracy	MCC
-1	98.52	15.47	57	0.25

-0.9	97.93	20.43	59.18	0.29

-0.8	96.55	25.2	60.87	0.31

-0.7	95.07	30.68	62.88	0.34

-0.6	93.27	36.96	65.11	0.37

-0.5	90.87	43.59	67.23	0.39

-0.4	88.44	50.1	69.27	0.42

-0.3	85.48	56.34	70.91	0.44

-0.2	82	63.34	72.67	0.46

**0**	**74.44**	**75.79**	**75.11**	**0.50**

***0.1***	***70.01***	***80.39***	***75.2***	***0.51***

0.2	65.41	84.4	74.9	0.51

0.3	60.32	87.78	74.05	0.5

0.4	55.85	90.6	73.23	0.5

0.5	51.22	92.97	72.09	0.49

0.6	46.39	94.58	70.48	0.47

0.7	40.21	96.12	68.17	0.44

0.8	34.63	97.44	66.03	0.41

0.9	28.65	98.03	63.34	0.37

1	21.78	98.92	60.35	0.33

(Italic-bold values indicate the point where sensitivity and specificity is roughly equal and Bold values indicate point where maximum Accuracy and MCC.)

### SVM Module based on physico-chemical parameters

In this study we have also developed SVM module using various physico-chemical features, which, are important for protein structure and function. Seven physico-chemical parameters have been used for this (see methods section). We have normalized these parameters [36] before using them for developing SVM classifier. Performance of SVM Module based on physico-chemical parameters is shown in Table 4. As shown in Table 4, performance (maximum MCC 0.26) was lower than SVM module based on sequence.

**Table 4:** The Performance of SVM model (Learning Parameter: g: 0.001 c: 4 j: 1) Using seven physiochemical properties.

Threshold	Sensitivity	Specificity	Accuracy	MCC
-0.9	93.68	18.71	56.19	0.19

-0.8	92.35	22.05	57.2	0.2

-0.7	90	26.85	58.43	0.22

-0.6	87.35	31.23	59.29	0.22

-0.5	84.3	36.72	60.51	0.24

-0.4	80.43	41.79	61.11	0.24

-0.3	76.32	47.35	61.84	0.25

-0.2	72.45	52.68	62.57	0.26

-0.1	68.25	57.78	63.01	0.26

**0**	**63.18**	**62.95**	**63.06**	**0.26**

0.1	57.95	68.08	63.01	0.26

0.2	52.55	72.75	62.65	0.26

0.3	47.15	77.05	62.1	0.25

0.4	41.56	80.86	61.21	0.24

0.5	36.56	84.77	60.66	0.24

0.6	31.79	87.42	59.6	0.23

0.7	26.99	89.57	58.28	0.21

0.8	23.15	92.55	57.85	0.22

0.9	19.44	94.34	56.89	0.21

(Bold values indicate point where maximum Accuracy and MCC.)

## Discussion

The ATP interacting proteins play a significant role in signaling pathways, in which ATP is used as a substrate by kinases that phosphorylate proteins. The identification of ATP interacting residues is difficult using experimental techniques. There is a need for developing computational techniques for identifying ATP interacting residues in a protein from its protein sequence. Saito et al [15] developed a general method for predicting binding site using empirical scores system. Though this method allows detection of ATP binding sites on a protein with low accuracy but provides no information about ATP interacting residues. There are methods, which allow identifying ATP interacting residues in a protein if its structure is known [19,37]. These methods are basically assignment method, which assign ATP interacting residues in a PDB file. In this study an attempt has been made to predict ATP interacting residue in a protein with high accuracy. One of the obvious question arise can we used existing techniques for predicting ATP interacting residues. First we used BLAST for predicting ATP interacting residues. As shown in result section we obtained poor performance both in terms of sensitivity and probability of correct prediction. Thus the routinely used similarity search technique like BLAST is not suitable for this problem. In the next step, we examine motif-based techniques for predicting ATP interacting residues. We search motifs using FingerPRINTScan [38] in 168 ATP binding protein chains used in this study and got motifs only in 54 proteins. No motif was found in the remaining 114 proteins. These motifs only cover around 11% ATP interacting residues (Table S2; see in Additional file) and no common motif was found in ATP binding protein (Table S3; see in Additional file). These results shows that motifs based method cannot be used for identifying of ATP interacting residues.

This study is a systematic attempt to understand and predict ATP interacting residues in a protein First we analyzed ATP interacting residues and its neighbors, and found that there is a significant difference in interacting and non-interacting residues. This means ATP interacting residues can be predicted using any machine leaning techniques. It has been shown in previous studies that SVM perform better than other artificial intelligence technique particularly on small dataset. Thus SVM based model has been developed for predicting ATP interacting residues in a protein from its primary structure and achieved reasonable accuracy. As PSSM based evolutionary information provide better information [9], hence we also made an attempt to develop method using evolutionary information for predicting ATP interacting residues. The performance of SVM module increases significantly when evolutionary information is in place of single sequence. This demonstrates that evolutionary information is important for predicting ATP interacting residues. In this study we used window size 17; the question arises why we have used 17. Though window size 17 is frequently used in prediction of secondary structure of interacting residues, it does not mean that window size 17 is applicable to each problem. One should try different window size in order to find out optimize window size for a given problem. We try various window sizes from 7 to 25 residues for predicting ATP interacting residues and achieved maximum performance for window size 17. Although accuracy of binary pattern of 25 window size is better than 17 but difference in sensitivity and specificity is much higher. This means that window size 17 is most suitable for predicting ATP interacting residues. This is first study of this kind so it is difficult to compare its performance with existing methods. We hope this study will be useful for researchers working in this area. There is a high probability that other researcher will work on this problem and will develop better method.

## Conclusion

In this study we have develop method, for the first time, for predicting ATP interacting residues in a protein from its protein sequence using SVM based model. It was observed that the evolutionary information (PSSM) based SVM modules perform better than the single sequence based modules. Though it has been shown in number of previous studies that the evolutionary information is important for predicting the structural component of a protein, first time we have demonstrated that the evolutionary information is also important for predicting ATP interacting residues. One of the major features of this study is that we are providing web service for predicting ATP interacting residues in a protein. Our web-server; ATPint allows users to identify ATP binding residue using the best model trained on our data set. This server will help the experimental biologist to predict ATP interacting residue from its primary sequence and avoid the number of essential experiments.

## Competing interests

The authors declare that they have no competing interests.

## Web Server

We developed a web server "ATPint" using CGI-Perl 5.8.4 script to predict of ATP interacting proteins, which is available at http://www.imtech.res.in/raghava/atpint. This server allows users to predict ATP interacting proteins using PSSM based SVM models. User can select any threshold within -1 to +1 by default it is 0.2. The prediction result presented in graphical form where the predicted ATP interacting and non-interacting are displayed in different color.

## Authors' contributions

JSC created dataset and developed the SVM models and NKM re-checked these models and datasets. JSC, NKM created the backend web server and the front end user interface. GPSR conceived the project, coordinated it and refined the final manuscript drafted by JSC and NKM. All authors have read and approved this manuscript.

## Supplementary Material

Additional file 1 **Table S1**. P-value for compositional difference in ATP interacting residue and non-interacting residue. Table S2, S3. Motifs based analysis. FprintScan AccNumber list and its frequency. Probability of percentage coverage of FprintScan in ATP interacting residue. Click here for file
