# Supplementary material for: Identification of ATP binding residues of a protein from its primary sequence
Source: BMC Bioinformatics. 2009 Dec 19;10:434. doi: 10.1186/1471-2105-10-434 (PMC2803200; doi:10.1186/1471-2105-10-434)
Supplement: Additional file 1 — Table S1. P-value for compositional difference in ATP interacting residue and non-interacting residue. Table S2, S3. Motifs based analysis. FprintScan AccNumber list and its frequency. Probability of percentage coverage of FprintScan in ATP interacting residue. [file 1471-2105-10-434-S1.DOC]

**Supplementary data**

**Identification of ATP binding residues of a protein from its primary sequence**

**Table S1**: P-value for compositional difference in ATP interacting residue and non-interacting residue

| Amino acid | Composition of ATP interacting residue | Composition of non-interacting residue | P-value |
| --- | --- | --- | --- |
| A | 5.33385749 | 8.47528428 | **0.00049382** |
| C | 0.49083791 | 1.01430207 | 0.04144007 |
| D | 7.10059583 | 6.41378646 | 0.22511424 |
| E | 6.02114823 | 7.85324604 | **0.00654934** |
| F | 3.69815902 | 4.31939497 | 0.40972657 |
| G | 11.4524566 | 6.83953232 | **0.00186245** |
| H | 3.20694672 | 2.19232374 | 0.07941769 |
| I | 5.46457645 | 5.39916348 | 0.93334488 |
| K | 7.98444637 | 5.92273461 | **0.0025405** |
| L | 6.15111839 | 9.48963983 | **0.00511538** |
| M | 2.55254966 | 2.61801611 | 0.87990049 |
| N | 4.09052983 | 3.69757068 | 0.3656477 |
| P | 2.71600184 | 5.00631131 | **0.00011265** |
| Q | 2.91229421 | 3.73014345 | 0.10078503 |
| R | 7.72204572 | 5.26849802 | **0.00861743** |
| S | 6.90510574 | 5.16960303 | 0.08751448 |
| T | 6.11886653 | 5.36669769 | 0.14904696 |
| V | 5.17072623 | 7.00293101 | 0.02253264 |
| W | 0.91604891 | 0.98146187 | 0.80861897 |
| Y | 3.99168833 | 3.23935903 | 0.17704468 |

**(**Bold values are significantly different in ATP interacting residue and non-interacting residue**)**

**Table S2**: FPrintScan based analysis of ATP interacting residues. In FPrintScan we use 168 ATP interacting protein sequences, out of these 54 proteins give small motif patterns, remaining 114 proteins have no any motiffs.

| **S. No.** | FPrintScan AccNumber | **No. of actual interacting residues** | **No. of interacting residues in FprintScan motif** | **No. of interacting residues in FPrintScan motif that common with actual interacting residues** |
| --- | --- | --- | --- | --- |
| 1 | PR01043 | 22 | 46 | 03 |
| 2 | PR01042 | 18 | 55 | 07 |
| 3 | PR01395 | 02 | 129 | 00 |
| 4 | PR01396 | 03 | 109 | 03 |
| 5 | PR01099 | 19 | 84 | 07 |
| 6 | PR00193 | 25 | 127 | 23 |
| 7 | PR00091 | 19 | 57 | 11 |
| 8 | PR01020 | 23 | 101 | 23 |
| 9 | PR01770 | 21 | 67 | 00 |
| 10 | PR00987 | 22 | 46 | 09 |
| 11 | PR00597 | 21 | 134 | 06 |
| 12 | PR01040 | 25 | 71 | 13 |
| 13 | PR01046 | 22 | 48 | 07 |
| 14 | PR01607 | 12 | 120 | 03 |
| 15 | PR00987 | 18 | 54 | 10 |
| 16 | PR00988 | 15 | 27 | 09 |
| 17 | PR00502 | 22 | 29 | 05 |
| 18 | PR00298 | 26 | 122 | 15 |
| 19 | PR00959 | 23 | 82 | 15 |
| 20 | PR01039 | 27 | 66 | 16 |
| 21 | PR01438 | 25 | 52 | 15 |
| 22 | PR00301 | 24 | 99 | 14 |
| 23 | PR01047 | 25 | 104 | 20 |
| 24 | PR01590 | 19 | 33 | 00 |
| 25 | PR01049 | 26 | 32 | 00 |
| 26 | PR00072 | 14 | 141 | 04 |
| 27 | PR01368 | 24 | 81 | 03 |
| 28 | PR01217 | 05 | 91 | 00 |
| 29 | PR00502 | 13 | 29 | 03 |
| 30 | PR00190 | 16 | 90 | 00 |
| 31 | PR00990 | 20 | 45 | 07 |
| 32 | PR00502 | 18 | 29 | 04 |
| 33 | PR00477 | 25 | 170 | 15 |
| 34 | PR01243 | 14 | 93 | 12 |
| 35 | PR01405 | 15 | 113 | 15 |
| 36 | PR00980 | 15 | 64 | 08 |
| 37 | PR01039 | 22 | 66 | 12 |
| 38 | PR00130 | 24 | 216 | 16 |
| 39 | PR00794 | 19 | 78 | 06 |
| 40 | PR00775 | 25 | 133 | 25 |
| 41 | PR00906 | 15 | 120 | 06 |
| 42 | PR01046 | 12 | 48 | 02 |
| 43 | PR00789 | 29 | 94 | 17 |
| 44 | PR01100 | 18 | 62 | 12 |
| 45 | PR01046 | 11 | 48 | 07 |
| 46 | PR01438 | 21 | 52 | 12 |
| 47 | PR01768 | 12 | 106 | 06 |
| 48 | PR00301 | 24 | 81 | 11 |
| 49 | PR00340 | 17 | 76 | 16 |
| 50 | PR01438 | 21 | 52 | 15 |
| 51 | PR00982 | 11 | 72 | 07 |
| 52 | PR00717 | 22 | 77 | 01 |
| 53 | PR01183 | 14 | 125 | 00 |
| 54 | PR00869 | 09 | 79 | 07 |
| Total | ----- | **1009** | **4425** | **483** |

**Average percentage coverage =** 483/1009*100

**=** 47.87%

**Probability of coverage Percentage =**483/4425*100

**=** 10.92%

**Table S3:** FprintScan AccNumbers which are repeated frequently (7 FprintScan AccNumbers are repeated only two or three times out of total 54 FprintScan AccNumber).

| **S. No.** | **FprintScan AccNumber** | **Frequency (repeated number)** |
| --- | --- | --- |
| 1 | PR00301 | 02 |
| 2 | PR00502 | **03** |
| 3 | PR00987 | 02 |
| 4 | PR01039 | 02 |
| 5 | PR01046 | **03** |
| 7 | PR01438 | **03** |

This table shows that there is no any common motif in ATP binding proteins, only few motifs observed which are common in maximum 3 proteins.
